# Effect of Supine Versus Sitting Positions on Facial Tissue Dielectric Constant Values in Young Adult Men

**DOI:** 10.7759/cureus.115349

**Published:** 2026-08-28

**Authors:** Harvey N Mayrovitz

**Affiliations:** 1 Medical Education, Nova Southeastern University Dr. Kiran C. Patel College of Allopathic Medicine, Davie, USA

**Keywords:** eczema, facial skin moisturization, psoriasis, rosacea, skin hydration, skin water, tdc, tissue dielectric constant

## Abstract

Background

Assessing facial skin epidermal and dermal water provides information about the skin’s water content, which is relevant to its barrier function and may be associated with conditions such as psoriasis, eczema, and rosacea. One method that permits quantitative assessment of facial skin dermal water is via measurements of the tissue dielectric constant (TDC), but such data are sparse in the literature, especially as it applies to assessment in men’s facial skin. The general lack of such data may limit the use of dermal water measurements to assess or track facial skin conditions. As an initial step toward improving this situation, this study aimed to evaluate facial TDC values in healthy adult males measured while supine and in the sitting position. These different positions were chosen to assess the potential impact of gravitational forces on the water content of facial tissue.

Methodology

TDC was measured bilaterally in triplicate to a 1.5 mm depth on the forehead, cheek, and jowl areas of 30 skin-healthy men after 15 minutes of supine lying and then after 15 minutes of upright sitting. Subject ages (mean ± SD) were 25.37 ± 2.59 years. TDC values on the right and left sides at corresponding sites were compared while the subjects were supine and seated. For each site, the right- and left-side TDC values were averaged and used to evaluate the changes associated with shifts in position. To account for the use of multiple right-left side comparisons, statistical acceptance of side differences was based on a p-value less than 0.01.

Results

While supine, left-right TDC values did not statistically differ at any site. The right-left average TDC values for forehead, cheek, and jowl (mean ± SD) were 41.38 ± 2.99, 39.24 ± 3.99, and 43.23 ± 4.13, respectively. While seated, all site average values were slightly lower: 40.36 ± 2.24, 38.76 ± 3.96, and 42.48 ± 3.47, but only the forehead difference was statistically significant (p = 0.001). Although statistically significant, the forehead difference was less than 1% and not likely to be of clinical significance.

Conclusions

For this group of young adults, facial skin TDC values were nearly symmetrical side-to-side, differed slightly among facial sites, and varied minimally between supine and sitting positions. If similar features exist in older men or in patients with the various skin conditions cited, then TDC values may be measured in either position, whichever is more convenient for the patient and examiner. Although the positional differences herein were small, further research into these differences in the presence of skin conditions is warranted. The currently determined TDC values, as indices of dermal water, may still serve as healthy-skin reference values for males that could make TDC measurements a useful modality for assessing and tracking changes in facial skin water across various skin conditions. However, the fact that the data from the present study were obtained via measurements on only 30 young adult men should be considered when applying the findings to other populations.

## Introduction

Assessing facial skin’s epidermal and dermal water content can provide useful information about the skin’s barrier function, which, if compromised, can make the skin more susceptible to inflammatory processes. Examples of conditions in which skin water is clinically deficient include psoriasis [1-7], eczema [8-12], and rosacea [13,14]. Skin measurements may, in some situations, help detect or track overall dehydration states. Methods for non-invasively estimating facial skin water content depend on which aspect of the skin tissue is of interest. One approach is based on estimating skin surface water, primarily that of the stratum corneum, by measuring the electrical capacitance or conductance of the epidermis at frequencies near 1 MHz [15-18], and has been applied to measuring facial properties [17]. Although useful in many contexts, one potential limitation of this method is that it provides no direct information on the water content of the underlying dermis. Another non-invasive method measures the skin tissue dielectric constant (TDC), which, depending on the probe size used, can provide information on dermal water by assessing skin water content [19-22]. Because this method operates at approximately 300 MHz, the TDC value is sensitive to both free and bound water in tissue, which is a distinct advantage, as some skin water is bound to proteins [23-25]. This method has been used to estimate facial dermal water in women [26,27], but there is sparse literature reporting men’s TDC facial values. The dominance of studies focused on women stems primarily from their importance in cosmetic contexts. However, as noted, various diseases prevalent in both women and men, such as rosacea, psoriasis, and eczema, are linked to reduced skin water. It would thus be useful to have information on normal facial skin dermal water values in men at various facial locations for potential comparative purposes. To obtain and provide such data, a decision must be made regarding the subject’s position during these facial measurements, because subsequent clinic-related measurements might be taken with patients in a supine or seated position, whichever is more appropriate for the patient and the examiner. This is considered a potential issue as it may be hypothesized that, due to gravity, supine skin water values would exceed those obtained with the patient seated. Thus, the specific aim of the present study was to obtain estimates of facial skin water in men while they were supine and seated.

## Materials and methods

Subjects

A total of 30 healthy male subjects, aged 21 to 35 years (mean ± SD = 25.37 ± 2.59 years), participated in this Institutional Review Board-approved research study (approval number: NSU 07300901F). Subjects had a wide body mass index (BMI) range from 19.6 to 45.2 kg/m², with a mean and SD of 25.14 ± 5.49 kg/m². Based on BMI classifications, four were obese, seven were overweight, and 19 had normal weight. Inclusion criteria for participation were males aged 21-35 years with no facial hair, no open facial sores, and no visibly apparent facial skin condition. Subjects were instructed to shave at least two hours before their scheduled appointment. All initially recruited subjects participated, arrived on time for their scheduled appointments, and none were excused for any reason.

Measurement method

The method is based on the principle that the TDC is directly related to the amount of free and bound water contained in the measuring volume [19-22], and at 300 MHz, the TDC is a direct measure of this water content. The TDC of specific target areas is determined using a coaxial probe that contacts the skin for about six seconds. The probe is handheld and connected via a coaxial cable to a control and processing unit with a display that indicates the TDC value (MoistureMeter-D, Delfin, Kuopio, Finland). The device generates a 300 MHz signal that is transmitted to the probe and into the tissue, whereupon a portion is reflected back to the control unit, where it is processed, and the TDC value is determined. The effective penetration depth depends on the probe size, with larger diameter probes penetrating deeper [28]. The output parameter is the TDC value, which ranges from 1 to 80. For comparison, pure water has a value of about 76 at 34°C. For the present study, a probe with a 20 mm diameter and an effective penetration depth of approximately 1.5 mm was used (Model S15, Delfin, Kupio, Finland). Skin TDC measurements have been widely used to assess skin water in arms [29,30] and legs [20-23] under normal and lymphedematous conditions [24-28] and are a widely accepted method.

Face measurement sites

**Figure 1:**
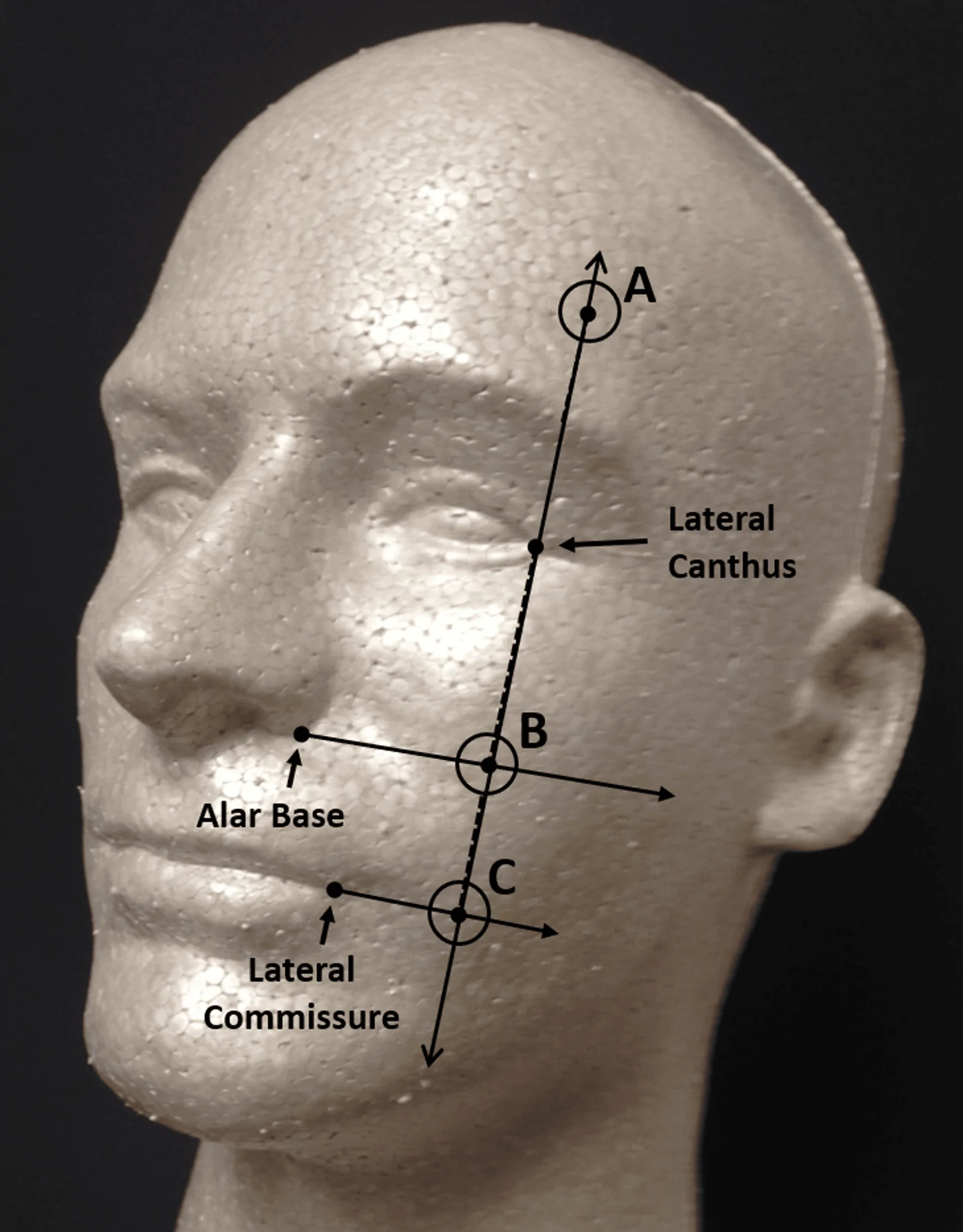
Face measurement sites. Tissue dielectric constant measurements were taken on the left and right sides of the face, along a vertical line adjacent to the lateral canthus of the eye, as schematically depicted in the figure for the left side. The forehead site (A) was 6 cm superior to the lateral canthus. Site B was determined by the intersection of the vertical line with a perpendicular from the nasal alar base. Site C was determined by the intersection of the vertical line with a perpendicular from the lateral commissure. This figure was created by the author by photographing a generic plastic model, then importing the image into Microsoft PowerPoint (Microsoft Corp., Redmond, WA, USA), and annotating it as shown.

Measurements were taken at specific sites on the left and right sides of the face along a vertical line adjacent to the lateral canthus of the eye, as schematically depicted in Figure 1 for the left side. The forehead site (A) was 6 cm superior to the lateral canthus. Site B on the cheek was determined by the intersection of a vertical line with a perpendicular line drawn from the nasal alar base. Site C was determined by the intersection of a vertical line with a perpendicular line starting from the lateral commissure, which approximated the jowl area. These sites were chosen as representatives of facial areas commonly assessed in physiological and pathophysiological studies of facial skin.

Protocol and measurement sequence

When a subject arrived for their appointment, they were asked if they had any questions or concerns about the study, and, if so, these were addressed. They then formally signed the Institutional Review Board-approved consent form. The subject was then asked to visit the restroom as needed. The subject then assumed a supine position on a padded examination table. While supine, the forehead, cheek, and jowl sites on the left side of their face were marked, and then the corresponding sites on the right side were marked. TDC measurements with the subject supine began after 15 minutes in the supine position. TDC measurements were first made on the left side of the face, starting with the forehead, progressing to the cheek, and then to the jowl area. This sequence was then repeated using the same order on the right side. This pattern was repeated twice more to obtain triplicate measurements at each of the six sites. The subject then assumed a seated position, and the complete measurement sequence was repeated, beginning after the subject had been sitting upright for 15 minutes. For each position (supine and sitting), the average of the triplicate TDC measurements at each site was used as the TDC value for that site. Room temperature (mean ± SD) at the start and end of the measurement procedures was 25.2 ± 1.2°C and 25.0 ± 1.1°C. Corresponding values for the room relative humidity were 35.8 ± 3.5% and 35.6 ± 3.7%.

Data analysis

The distribution of triplicate TDC average values among subjects at each site was tested for normality using the Shapiro-Wilk test; a p-value >0.05 indicated normality. These test results indicated no deviation from normality. Differences in TDC values between face sides at corresponding anatomical locations were assessed using paired t-tests. To account for the use of multiple right-left comparisons, statistical significance of side differences was defined as a p-value <0.01. Assessments of differences between supine and seated TDC values were based on comparisons of the averages of the left and right TDC values for each site, the forehead (Site A), cheek (Site B), and jowl areas (Site C), with a p-value <0.01 indicating a statistically significant difference. Tests for differences among facial sites were based on a general linear model for repeated measures, with site-average TDC values as the within-subject variable. An estimate of the repeatability of the triplicate TDC measurements was obtained by computing the coefficient of variation from the left- and right-side values for each site, calculated when the subject was supine and seated.

## Results

Individual site and posture-related tissue dielectric constant values

**Figure 2:**
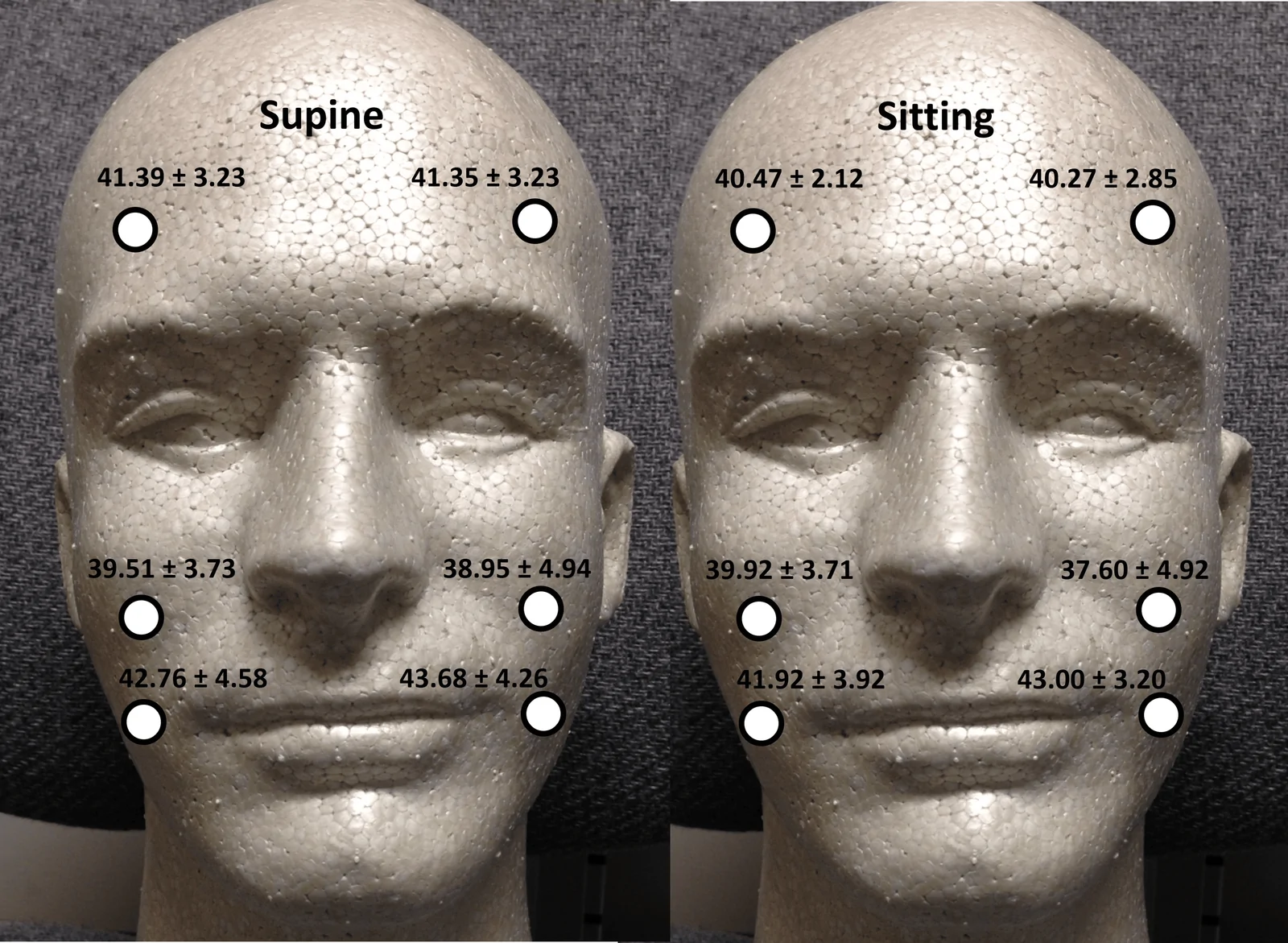
Facial tissue dielectric constant (TDC) values. The values indicated are the mean ± standard deviation of TDC measurements at each site, obtained from subjects initially supine and then in an upright seated position. There were no statistically significant differences in average TDC values between sides while subjects were supine or sitting. This figure was created by the author by photographing a generic plastic model, then importing the image into Microsoft PowerPoint (Microsoft Corp., Redmond, WA, USA), and annotating it as shown.

Figure 2 summarizes the TDC values for each facial site measured after the subject was supine for 15 minutes and then after the subject was in an upright seated position for 15 minutes. There were no statistically significant differences in average TDC values between the right and left facial sides when subjects were supine or sitting, indicating a reasonable degree of facial-side symmetry in dermal water content. As indicated in Figure 2, the maximum variation in the average TDC values among sites in the supine position was approximately 11.5%, with the mean TDC values ranging from a low value of 38.95 at the left cheek to a high value of 43.68 at the left jowl area. In the seated position, the mean TDC values ranged from a low value of 37.60, also on the left cheek, to a high value of 43.00 at the left jowl area. This amounts to a 13.4% difference. Based on the outcome of the general linear model with repeated measures, the TDC values among the forehead, cheek, and jowl were statistically significant (p < 0.001) in both the supine and sitting positions. Based on the outcome of the general linear model with repeated measures, there was an overall statistically significant difference in the average TDC values among the forehead, cheek, and jowl (p < 0.001) in both the supine and sitting positions, with each site statistically different from the others.

Effects of body position on facial tissue dielectric constant values

The right- and left-side TDC values were averaged to produce a single average TDC value representing each of the three facial regions that were evaluated for the supine and the seated positions. Table 1 summarizes these results. The only statistically significant difference in TDC values between subjects supine and seated was at the forehead, where the seated value was slightly lower than the supine value (40.36 ± 2.24 vs. 41.38 ± 2.99, p = 0.002). The average TDC value for each of the other sites was slightly lower when measured with the subject seated, but these differences were not statistically significant. The standard deviation of TDC values among subjects was least at the forehead and greatest at the cheek. The percentage difference between supine and sitting TDC values for each subject was calculated as the difference between the supine and seated positions, divided by their average, multiplied by 100. The average of these 30 individual differences, along with the associated standard deviations, was then determined. Values corresponding to the forehead, cheek, and jowl were 0.62 ± 1.00%, 0.31 ± 2.32%, and 0.44 ± 1.49%.

**Table 1:** Tissue dielectric constant (TDC) values by subject body position during measurement. Values are the TDC mean ± SD of the averaged right and left face sides for each site. P-values are based on a paired t-test, with p-values <0.01 considered statistically significant.

	Subject Position		
Site	Supine	Seated	P-Value
Forehead	41.38 ± 2.99	40.36 ± 2.24	0.002
Cheek	39.24 ± 3.99	38.76 ± 3.96	0.452
Jowl	43.23 ± 4.13	42.48 ± 3.47	0.109

Repeatability of tissue dielectric constant measurements by site and position

The coefficient of variation (CV) was determined as the ratio of the standard deviation to the mean value of all TDC measurements at a given site for each position. When triplicate measurements were made with subjects in a supine position, the CV was lowest (best repeatability) at the forehead (2.27 ± 2.02%), and highest (least good repeatability) at the cheek (4.22 ± 3.74%), with measurements at the jowl area having a CV in between at 3.76 ± 3.46%. The forehead CV was statistically significantly lower than that at the cheek (p < 0.001). When the subject was in the seated position, the CV was also lowest at the forehead (3.55 ± 2.84%) and highest (most variable) at the cheek site (4.69 ± 4.33%), with the jowl site CV in between at 3.67 ± 3.28%. However, as can be seen from the metrics, no site exceeded a mean of 5%, which is consistent with good reproducibility for in vivo measurements of this type.

## Discussion

Main findings

The findings indicate that the facial skin TDC values of this population of young adult skin-healthy men were nearly symmetrical side-to-side and differed slightly among facial sites. For a given site (forehead, cheek, or jowl), TDC values varied negligibly with body position between supine and upright sitting. The only position-related difference that demonstrated statistical significance was at the forehead. However, the difference was less than 1% and within the limits of the measured CV and not clinically relevant. Although that difference was less than 1% and therefore clinically negligible, it might be explained by the gravitational drainage associated with this most superior facial site. The directional change is consistent with the initial hypothesis of a potential gravitational impact, but the magnitude, as noted, is negligibly small.

The potential clinical implications of the findings are twofold. First, it provides preliminary reference values for expected TDC values and their variation among healthy subjects. This may allow assessment of the normality of TDC values measured in a similar manner in patients with suspected or known facial skin conditions, including rosacea [31], psoriasis [32], or eczema [33]. However, these proposed applications remain hypothetical and require validation in disease populations. Second, the findings show that patients may be measured while they are supine or sitting, whichever is more convenient for the patient and examiner, with negligible differences in resultant TDC values. The present findings also have implications for our understanding of fundamental aspects of skin physiology and for the utilization of skin water assessments in patients with suspected or overt facial skin conditions. However, it is emphasized that because the present findings are specific to young, skin-healthy subjects, the extension to older subjects and those with the various skin conditions noted remains to be determined. This qualification applies to both the measured TDC values and the extent to which differences in position may affect them. However, the present data provides important baseline reference ranges for both of these aspects for comparisons.

Considerations as to measurement depth

As noted, the effective measurement depth of the currently used TDC probe was 1.5 mm. This depth was specifically chosen to primarily assess the combined epidermal and dermal water content, with minimal contribution from underlying subcutaneous fat. Although the facial skin depth was not measured in the present study, skin depth values have been reported for multiple skin sites based on assessments using very high frequency ultrasound at 75 MHz [34]. An examination of these authors’ face maps indicated that the skin depth of the lateral forehead, cheek, and jowl regions, as measured herein, had median depths (epidermis + dermis) of 1.492 mm, 1.445 mm, and 1.367 mm, respectively. Given these dimensions, it is clear that the present TDC measurements would, in fact, mainly reflect dermal water content. Although the above skin depth measurements were performed on females, it is to be expected that male skin depths at corresponding sites would be somewhat greater based on the comparisons of male versus female face measurements using 24 MHz ultrasound measurements [35]. This comparison further supports the idea of little involvement of subcutaneous fat contributing to the present TDC measurements. This is important because fat has a low water content and a low TDC value [36,37]. If it were included in the measurement volume, it would artificially reduce the measured TDC value and yield inappropriately low TDC values for facial skin TDC.

Considerations as to absolute facial tissue dielectric constant values

It is of interest to compare the presently determined facial TDC values with those obtained in other anatomical areas. Given the effective measurement depth issue, the only valid comparisons would be those in which TDC was measured to a depth of 1.5 mm or less, as otherwise the hypodermis contribution would be included. This transition has been well demonstrated with TDC measurements made at varying depths at the same anatomical site, such as the volar forearm, where TDC values reported at a depth of 1.5 mm (32.8 ± 5.3) were reduced to 22.5 ± 3.9 at an effective depth of 2.5 mm [38]. However, there was little difference in TDC values measured at an effective depth of 0.5 mm, consistent with these measurements reflecting tissue volumes that are mainly dependent on dermal water content. At a measurement depth of 1.5 mm, similar TDC values have been reported for the forearm of 69 subjects: 31.8 ± 5.4. Thus, it can be reasonably concluded that facial skin TDC values, as measured herein, which ranged from approximately 39 to 43, depending on the specific site and posture, are greater than those of the forearm. This indicates greater facial water content than is otherwise present in forearm skin. Other anatomical site differences in skin water based on TDC measurements at 1.5 mm have been demonstrated to be present in both males and females [39]. In that study of 50 males and 50 females, TDC values ranged from approximately 27-29 at the ankle, 32-37 at the forearm, and 37 on the anterior chest. TDC differences between facial and forearm skin may be influenced by the reported thicker cheek skin compared with forearm skin [40]. These prior observations emphasize the importance of accounting for the anatomical dependence of skin tissue water content in any comparative assessment, whether research- or clinically related.

Considerations as to gender

Although the currently determined absolute TDC values strictly apply to males, the finding of negligible postural variation may apply more generally to females. However, as there is considerable data supporting the observation that the skin thickness of males is greater than that of females [41,42], there remains a question as to how well the present absolute TDC values may differ from those that would be measured in females. Although there are no useful comparative data for facial TDC measurements at 1.5 mm depths, an estimate of the differential may be extrapolated from male-female TDC values obtained on the forearm skin of 150 males and 150 females [43]. In that study, male TDC values in a population of a similar age to the present population exceeded female values by an average of 13%, which the authors attribute, in part, to differences in skin thickness, as inferred from a model analysis comprising multiple skin layers.

Potential skin condition applications for future consideration

As far as can be determined, there have been no reports of the application of TDC measurements to aid in the management of specific facial skin conditions such as rosacea, psoriasis, or eczema. Given the simplicity of the measurement used to track skin water, the question arises as to whether its application for this purpose is warranted. In the case of rosacea, the literature is replete with studies that have evaluated the effects of various treatments on facial skin water, in which skin surface water is measured based on its electrical capacitance. One study of 20 patients with rosacea demonstrated an increase in this surface water with topical treatment that was accompanied by a parallel decrease in facial skin erythema and transepidermal water loss [44]. Although such tracking procedures are useful, the important aspects of the changes in the deeper dermal water are not known because of the depth limitation.

Similar considerations may also apply to treatment evaluations in patients with psoriasis in whom skin dryness is a feature that, if mitigated, may forestall treatment and help avoid recurrence. Evaluations of a small group of patients with psoriasis demonstrated that aquaporin expression was decreased compared with that in healthy controls and that stratum corneum surface capacitance decreased in parallel with an increase in transepidermal water loss [1]. However, the associated changes in the dermis of these patients remain unknown, a gap that could potentially be addressed by adding TDC measurements to investigational or treatment-related studies. The potential advantage of supplementing various treatment strategies with TDC measurements to clarify the effects of treatment on dermal water is evident in more recent studies related to the treatment of rosacea. Treatment with pulsed light therapy, with or without collagen dressings, in groups of 75 rosacea patients demonstrated the benefit of the combined therapy, in parallel with higher stratum corneum water levels and improvements in other parameters [45]. However, corresponding changes in dermal water are unknown. These potential applications extend to newer treatments, including those that address rosacea patients in whom standard treatments need to be supplemented. In one such pilot study, botulinum toxin proved effective and was associated with increased skin surface water, although its effect on dermal water remains unknown [46]. Although most recent quantitative studies evaluating the efficacy of various new treatments for rosacea include measures of stratum corneum water indices and often transepidermal water loss [47-49], none have examined the effects of these treatments on dermal water, thereby potentially missing important pathophysiological information regarding skin-related effects. These aspects would also apply to varying degrees to psoriasis of the face [50,51] and eczema [52-54].

Study limitations

One limitation of the present study is that the data were obtained from healthy young adults, so it is unclear what potential differences in values might exist in older healthy subjects or in patients with any noted skin conditions. This limitation may affect the absolute TDC values and the effect of different body positions on those values. Another potential limitation that may affect the absolute TDC values in the two positions concerns the fixed order in which the positions were changed, always from supine to sitting. The possibility that small observed differences could be attributed to temporal changes cannot be excluded. Differences in absolute TDC values that may be affected by differences in subject ethnicity, skin thickness, or BMI were not evaluated. Despite these limitations, the findings provide initial reference ranges for both of these aspects for subsequent comparisons.

## Conclusions

The overall findings indicate that the facial skin TDC values of this population of young adult, skin-healthy men are nearly symmetrical side-to-side but differ slightly among facial sites and, for a given site, vary negligibly with body position during measurements in supine or upright sitting. This finding indicates that in healthy young men, TDC measurements obtained supine or seated showed minimal differences that are unlikely to be clinically meaningful, so that subjects may be measured supine or sitting, whichever is more convenient for the patient and examiner, with negligible differences in the resulting TDC values. These should be viewed as preliminary data regarding positional effects. Furthermore, the currently determined TDC values, as indices of water in male facial skin tissue, serve as preliminary reference data that could make TDC measurements a useful modality for assessing and tracking changes in facial skin water across various skin conditions. However, the utility of such measures in conditions such as rosacea, psoriasis, or eczema remains to be determined and is a target well-suited for future research. A possible limitation of the present study is that the data are from younger men, and it remains unclear to what extent they fully represent older men or those with the cited skin conditions. Thus, further research on such populations is warranted and encouraged.
